# Risk of head and neck cancer in relation to blood inflammatory biomarkers in the Swedish AMORIS cohort

**DOI:** 10.3389/fimmu.2023.1265406

**Published:** 2023-10-09

**Authors:** Yanping Yang, Yushan Liang, Fatemeh Sadeghi, Maria Feychting, Niklas Hamar, Fang Fang, Zhe Zhang, Qianwei Liu

**Affiliations:** ^1^ Department of Otolaryngology-Head & Neck Surgery, First Affiliated Hospital of Guangxi Medical University, Nanning, Guangxi, China; ^2^ Key Laboratory of Early Prevention and Treatment for Regional High-Frequency Tumor (Guangxi Medical University), Ministry of Education, Nanning, China; ^3^ Guangxi Key Laboratory of High-Incidence-Tumor Prevention & Treatment (Guangxi Medical University), Nanning, China; ^4^ Institute of Environmental Medicine, Karolinska Institutet, Stockholm, Sweden

**Keywords:** inflammatory biomarkers, head and neck cancer, cohort study, nested case-control study, epidemiology

## Abstract

**Background:**

Inflammation is critically involved in the development of human cancer, and blood inflammatory biomarkers have been proposed to indicate the risk of different cancer types.

**Methods:**

Using the Swedish Apolipoprotein-Related Mortality Risk (AMORIS) Cohort (N=812,073), we first performed a time-to-event analysis to evaluate the association of the baseline level of 12 blood inflammatory biomarkers measured during 1985-1996 with the subsequent risk of head and neck cancer (HNC) identified through the nationwide Swedish Cancer Register until end of 2020. A nested case-control study was further conducted to demonstrate the longitudinal trends of the studied biomarkers during the 30-year period prior to diagnosis of HNC.

**Results:**

In the time-to-event analysis, we identified a total of 2,510 newly diagnosed HNC cases. There was an increased risk of HNC per standard deviation (SD) increase of haptoglobin (hazard ratio [HR]: 1.25; 95% confidence interval [CI]: 1.21-1.30), leukocytes (HR: 1.22; 95%CI: 1.17-1.28), sedimentation rate (HR: 1.17; 95%CI: 1.07-1.29), and monocytes (HR: 1.34; 95%CI: 1.07-1.68) at baseline, after adjustment for age, sex, fasting status, occupational status, and country of birth. In contrast, there was a decreased risk of HNC per SD increase of lymphocytes in % (HR: 0.85; 95%CI: 0.73-0.99) and lymphocyte-to-monocyte ratio (LMR) (HR: 0.81; 95%CI: 0.69-0.95) at baseline. In the nested case-control study using repeatedly measured biomarker levels, we found that individuals with HNC had consistently higher levels of haptoglobin, leukocytes, sedimentation rate, and monocytes, as well as consistently lower levels of lymphocytes in % and LMR, during the 30-year period prior to diagnosis, compared to controls.

**Conclusion:**

Based on a cohort of more than half a million participants with up to 35 years of follow-up, our findings provide solid evidence supporting the presence of alterations in blood inflammatory biomarkers during the decades before diagnosis of HNC.

## 1 Introduction

In 2020, head and neck cancer (HNC) ranked as the 7^th^ most prevalent cancer globally, comprising 4.8% of all incident cancer cases and 4.7% of all cancer deaths (1). HNC includes cancers in the lip and oral cavity, salivary glands, nose, middle ear, pharynx, and larynx, most of which are squamous cell carcinomas (2). In addition to smoking and alcohol use, exposure to betel quid, wood dust, radiation, and genetic risk factors is also associated with the risk of HNC (2). HPV infection has also been identified as a risk factor for HNC, particularly squamous cell carcinoma (2). During the last decades, a decreasing incidence and mortality of HNC have been noted, likely because of decreasing tobacco use (3, 4).

Cancer-related inflammation is one of the seven hallmarks of cancer (5, 6). Cancer can promote the expression of inflammation-related factors such as interleukins and chemokines (7), whereas inflammation can promote cancer progression by regulating the immune microenvironment through a cascade of inflammatory factors such as cytokines and infiltrating leukocytes (8). Many studies have therefore examined the roles of different inflammatory biomarkers (e.g., haptoglobin, C-reactive protein [CRP], albumin, platelets, sedimentation rate, leukocytes, lymphocytes, monocytes, and neutrophils) in the risk of different cancers, including HNC (9–50). Different mechanisms might underlie the link between these biomarkers and cancers. For instance, metabolic dysfunction has been proposed as a potential pathway linking together altered expression of haptoglobin and different malignancies (51, 52) whereas a high level of sedimentation rate might indicate presence of inflammation and tissue damage in the body, which could subsequently influence the risk of cancers (37, 53). Similarly, the profiles of different immune cells have been studied extensively in cancer development and progression, as immune system function is critically involved in the initiation and progression of cancers (54). Although most of the studies found a difference in inflammatory biomarkers between patients with HNC and individuals free of HNC, there is a concern of potential reverse causation as most of these studies are case-control studies with biomarker measurements after a diagnosis of HNC. Large-scale prospective studies are therefore needed to confirm or refute these findings. Furthermore, few studies have examined HNC by histopathology type or cancer site or examined a comprehensive panel of inflammatory biomarkers commonly measured clinically.

To this end, we conducted several analyses using the Swedish Apolipoprotein-Related Mortality Risk (AMORIS) Cohort, including a sample size of over half a million and a follow-up of up to 35 years, with the aim of evaluating the association between the baseline level of blood inflammatory biomarkers that are commonly measured clinically (e.g., CRP, haptoglobin, albumin, platelets, sedimentation rate, and counts as well as frequencies of immune cells) and the subsequent risk of HNC, focusing on analyzing HNC by histopathology type and cancer site. We also performed a nested case-control study to examine the temporal trends of these biomarkers during the three decades prior to diagnosis of HNC.

## 2 Materials and methods

### 2.1 Study design

The Swedish AMORIS Cohort includes information on laboratory tests of blood and urine samples from health examinations in relation to an occupational health check-up or an outpatient visit in occupational or primary care from 812,073 individuals between 1985 and 1996 (55). Most of the participants in the AMORIS Cohort came from Stockholm, and the Central Automation Laboratory (CALAB) in Stockholm performed all laboratory analyses. The cohort has been followed from enrolment to December 31, 2020, via linkages to various Swedish national registers, including the Cancer Register, the Patient Register, the Causes of Death Register, consecutive Swedish Censuses (1970-1990), the Longitudinal Integration Database for Health Insurance and Social Market Studies (LISA) (1990 onward), and the Total Population Register, using the Swedish 10-digit personal identity number (55, 56). In the present study, we first performed a time-to-event analysis by following participants from their first blood sampling, where at least one of the studied biomarkers had a test result, i.e., baseline, until a diagnosis of HNC, emigration from Sweden, death, or December 31, 2020, whichever came first. Individuals who were younger than 20 at baseline (N=24,520) or had a previous diagnosis of cancer (N=12,963) were excluded, leaving 542,433 participants in the analysis.

We studied 12 blood inflammatory biomarkers, namely haptoglobin, CRP, albumin, platelets, leukocytes (i.e., granulocytes, lymphocytes and monocytes), sedimentation rate, lymphocytes, monocytes, neutrophils, lymphocytes in %, monocytes in %, and neutrophils in %. Total levels of haptoglobin and CRP were measured with an immunoturbidimetric technique. The automated Hitachi-analyzer was used to measure haptoglobin, while CRP was measured using fully automated multichannel analyzers (57). A sensitive quantitative method, the bromocresol green method, was used for the determination of serum albumin (58). Leukocytes, lymphocytes, monocytes, and neutrophils were routinely analyzed using hematology analyzers (Coulter STKS) (58). We subsequently calculated lymphocytes in %, monocytes in %, and neutrophils in %. The coefficient of variation was 5.6% for haptoglobin at a level of 1.1 g/L, <2.7% for leukocytes at a level of 10×10^9^/L, and 12% for CRP at a level of 40mg/L. We also calculated four ratios, namely neutrophil-to-lymphocyte ratio (NLR), platelet-to-lymphocyte ratio (PLR), lymphocyte-to-monocyte ratio (LMR), and CRP-to-albumin ratio (CAR). It is worth noting that, although these ratios are derived from specific immune cells, CRP, and albumin, they are known to provide complementary information, e.g., NLR reflects dynamic relationship between innate (e.g., neutrophils) and adaptive (e.g., lymphocytes) cellular immune response during illness and various pathological states (59, 60). The date and fasting status of each measurement, as well as age and sex of the participants, were extracted from the AMORIS Cohort. From the Swedish Censuses in 1970, 1980, 1985, and 1990 as well as LISA, information on occupational status and country of birth was obtained.

The outcome of the study was a new diagnosis of HNC during follow-up, as identified through the Swedish Cancer Register, which has since 1958 collected nationwide data on newly diagnosed cancer cases in Sweden. The 9^th^ and 10^th^ Swedish revisions of the International Classification of Disease (ICD-9 and ICD-10) codes were applied to identify HNC cases. We classified HNC into squamous cell carcinoma and adenocarcinoma based on histopathological codes as well as by cancer site (i.e., cancer in the lip and oral cavity, cancer in the salivary glands, pharynx cancer, cancer in the nose and middle ear, and larynx cancer).

### 2.2 Statistical analysis

#### 2.2.1 Time-to-event analysis

A time-to-event analysis was conducted for each biomarker, utilizing the baseline measurement of the biomarker as the exposure of interest. Cox models were employed to calculate the hazard ratio (HR) and 95% confidence interval (CI) to estimate the association between biomarker levels and the risk of HNC. The models were adjusted for age, sex, fasting status, occupational status, and country of birth. The underlying time scale was attained age, and the initial five years of follow-up were excluded from the analysis to prevent potential reverse causality (i.e., blood biomarker levels might be secondary to the upcoming HNC).

We first analyzed the biomarkers as continuous variables, estimating the effect of each standard deviation (SD) increase. We then analyzed the biomarkers as quartiles, estimating the effect of each quartile increase. Finally, we used clinical references of the biomarkers to classify the study participants, comparing the risk of HNC among individuals with normal versus abnormal levels of the biomarkers. We first analyzed any HNC and then analyzed HNC by histopathological type and cancer site. To check for potential confounding by indication, which means that biomarkers measured in relation to a referral by an outpatient hospital visit might be affected by the reasons for the hospital visit, we did the main analyses again, this time limiting the analysis to baseline measurements of biomarkers taken during an occupational health check-up (i.e., screening).

#### 2.2.2 Nested case-control study analysis

As participants of the AMORIS Cohort could have more than one measurement of the studied biomarkers during the enrollment period, based on the study cohort, we also performed a nested case-control study to investigate the temporal trends of the biomarkers during the 30-year period prior to HNC diagnosis, considering both the baseline and all subsequent measurements of the biomarkers. Cases were identified as participants who were diagnosed with HNC during the follow-up of the study cohort. 25 controls were randomly selected from the cohort for each case according to the method of incidence density sampling (61) and individually matched to the case by age, sex, and calendar period of enrollment in the AMORIS Cohort. The index date was set as the date of diagnosis for cases and their controls. All available biomarker test results during the 30-year period prior to the index date were analyzed.

We first plotted the mean concentrations of the studied biomarkers for cases and controls over the 30-year period prior to the index date using locally weighted scatterplot smoothing. We performed this analysis for any HNC and squamous cell carcinoma but not adenocarcinoma due to the limited number of cases of the latter. Additionally, we used conditional logistic regression to calculate the odds ratio (OR) with 95%CI for any HNC by comparing the abnormal to normal levels of the biomarkers during the 15 two-year time windows prior to the index date. The matching set was used as the stratum indicator in this model, and the model was adjusted for fasting status.

The statistical analyses were performed using SAS version 9.4 (SAS Institute, Cary, NC), R software version 4.2.2, and Stata version 16.1 (StataCorp, Texas, USA). The significance level was set at p<0.05 using a two-tailed distribution.

## 3 Results

The study cohort included 542,433 participants with a mean age of 45.03 at baseline (**Table 1**). During a mean follow-up of 20.36 years, we identified a total of 2,510 newly diagnosed HNC cases.

**Table 1:** Characteristics of the study participants at baseline.

Characteristics	Entire cohort (N=542,433)	Men (N=284,841)	Women (N=257,592)
**Age, mean (SD)**	45.03 (14.42)	44.36 (13.56)	45.77 (15.27)
Country of birth, N (%)			
Sweden	45,9823 (84.77%)	245,562 (86.21%)	214,261 (83.18%)
Other Nordic countries	35,576 (6.56%)	15,617 (5.48%)	19,959 (7.75%)
Other	47,034 (8.67%)	23,662 (8.31%)	23,372 (9.07%)
Occupational status, N (%)			
Employed	461,642 (85.11%)	252,412 (88.62%)	209,230 (81.23%)
Unemployed	80,791 (14.89%)	32,429 (11.38%)	48,362 (18.77%)
Biomarkers, mean (SD)			
Haptoglobin in g/l (N=407,279)	1.07 (0.34)	1.07 (0.35)	1.07 (0.32)
CRP in mg/l (N=344,690)	6.06 (17.09)	6.17 (18.36)	5.95 (15.58)
Albumin in g/l (N=492,421)	43.20 (2.89)	43.81 (2.87)	42.53 (2.77)
Platelet in 10^9/l (N=194,827)	262.87 (67.02)	250.23 (63.57)	272.51 (67.98)
Leukocytes in 10^9/l (N=203,936)	6.63 (2.38)	6.61 (2.65)	6.65 (2.15)
Sedimentation rate in mm/hour (N=89,256)	8.90 (10.27)	7.00 (9.53)	10.34 (10.57)
Lymphocytes in 10^9/l (N=53,529)	2.25 (2.04)	2.30 (2.39)	2.22 (1.76)
Monocytes in 10^9/l (N=53,526)	0.41 (0.38)	0.43 (0.52)	0.39 (0.23)
Neutrophils in 10^9/l (N=27,961)	3.98 (1.78)	3.89 (1.74)	4.04 (1.80)
Lymphocytes in % (N=54,130)	33.70 (10.05)	34.13 (10.28)	33.40 (9.87)
Monocytes in % (N=54,127)	6.04 (3.03)	6.35 (3.23)	5.82 (2.86)
Neutrophils in % (N=28,203)	57.48 (10.36)	56.63 (10.52)	58.05 (10.20)
LMR^a^ (N=52,738)	2.59 (0.99)	2.53 (1.02)	2.62 (0.97)
PLR^a^ (N=49,805)	6.97 (0.60)	6.88 (0.62)	7.03 (0.58)
NLR^a^ (N=27,960)	0.89 (0.74)	0.85 (0.77)	0.91 (0.72)
CAR^a^ (N=328,928)	-3.66 (1.38)	-3.69 (1.40)	-3.62 (1.34)

a Logarithmic transformation (log2) was used for LMR, PLR, NLR, and CAR.

CRP, C-reactive protein; LMR, lymphocyte-to-monocyte ratio; PLR, platelet-to-lymphocyte ratio; NLR, neutrophil-to-lymphocyte ratio; CAR, C-reactive protein-to-albumin ratio.

### 3.1 Time-to-event analysis

**Figure 1:**
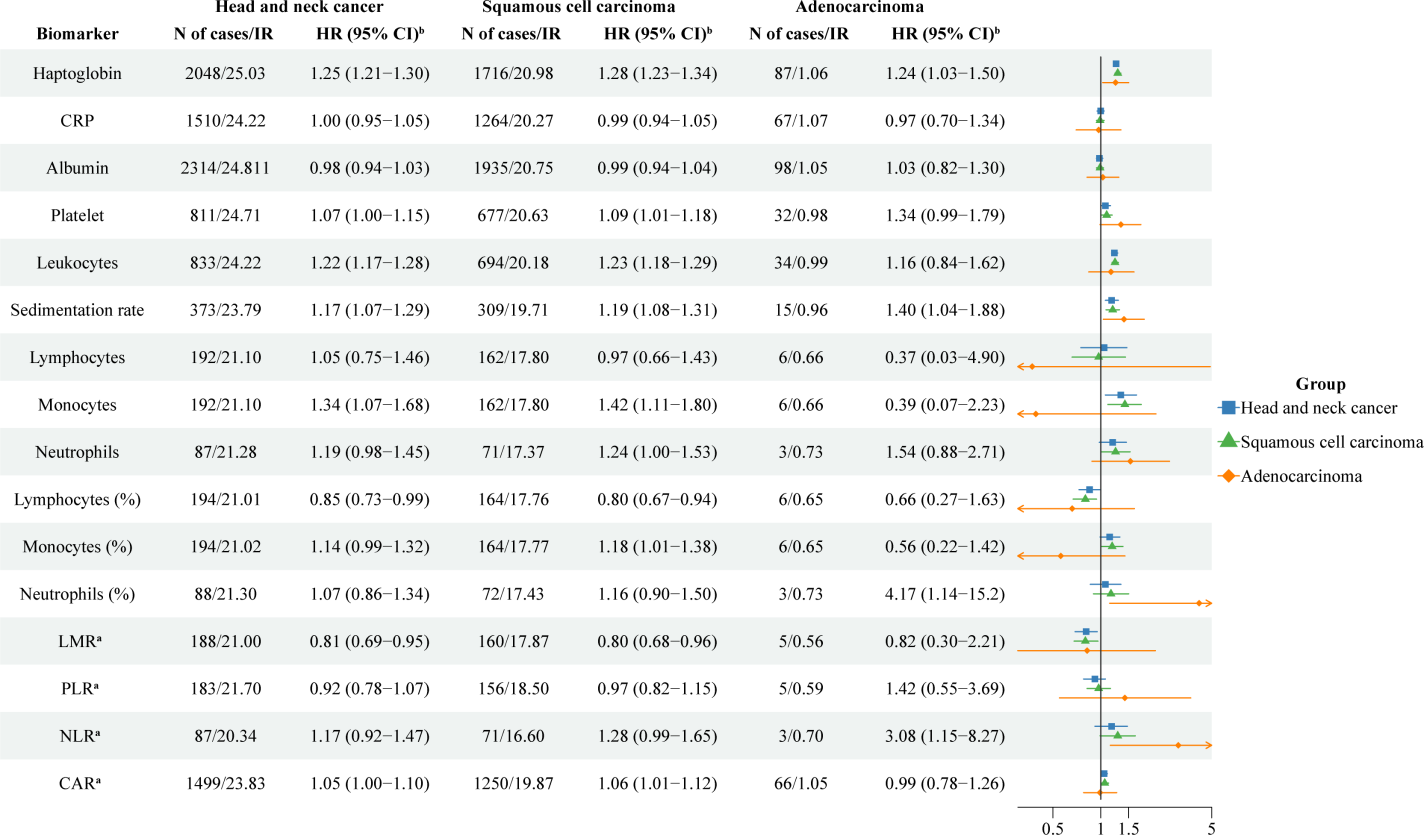
The association between one standard deviation increase of blood inflammatory biomarkers and risk of head and neck cancer. CRP: C-reactive protein; LMR: lymphocyte-to-monocyte ratio; PLR: platelet-to-lymphocyte ratio; NLR: neutrophil-to-lymphocyte ratio; CAR: C-reactive protein-to-albumin ratio; IR: Incidence rates; HR: hazard ratios; CI: confidence intervals. ^a^ Logarithmic transformation (log2) was used for LMR, PLR, NLR, and CAR. ^b^ Analyses were adjusted for age, sex, fasting status, occupational status, and country of birth.

The risk of HNC was positively associated with a higher baseline level of haptoglobin (HR: 1.25; 95%CI: 1.21-1.30 per SD increase), leukocytes (HR: 1.22; 95%CI: 1.17-1.28 per SD increase), sedimentation rate (HR: 1.17; 95%CI: 1.07-1.29 per SD increase), and monocytes (HR: 1.34; 95%CI: 1.07-1.68 per SD increase) after multivariable adjustment (**Figure 1**). However, the risk of HNC was negatively associated with a higher baseline level of lymphocytes in % (HR: 0.85; 95%CI: 0.73-0.99 per SD increase) and LMR (HR: 0.81; 95%CI: 0.69-0.95 per SD increase). The associations for haptoglobin and sedimentation rate were observed for both squamous cell carcinoma and adenocarcinoma. The associations for leukocytes, monocytes, lymphocytes in %, and LMR were only observed for squamous cell carcinoma. For squamous cell carcinoma, a statistically significant positive association was also noted for platelets (HR: 1.09; 95%CI: 1.01-1.18 per SD increase), monocytes in % (HR: 1.18; 95%CI: 1.01-1.38 per SD increase), and CAR (HR: 1.06; 95%CI: 1.01-1.12 per SD increase). For adenocarcinoma, a positive association was noted for neutrophils in % (HR: 4.17; 95%CI: 1.14-15.2 per SD increase) and NLR (HR: 3.08; 95%CI: 1.18-8.27 per SD increase). The results did not alter greatly when we restricted the analyses to measurements obtained during an occupational health check-up (**Figure S1**).

When analyzing HNC by cancer site, we found an increased risk of cancer in the lip and oral cavity per SD increase in haptoglobin (HR: 1.21; 95%CI: 1.14-1.29), platelets (HR: 1.12; 95%CI: 1.01-1.24), leukocytes (HR: 1.17; 95%CI: 1.08-1.28), and sedimentation rate (HR: 1.18; 95%CI: 1.02-1.35). We also found an increased risk of pharynx cancer per SD increase in haptoglobin (HR: 1.33; 95%CI: 1.24-1.42), leukocytes (HR: 1.28; 95%CI: 1.19-1.36), sedimentation rate (HR: 1.23; 95%CI: 1.04-1.46), neutrophils (HR: 1.57; 95%CI: 1.20-2.04), neutrophils in % (HR: 2.10; 95%CI: 1.30-3.41), and NLR (HR: 2.23; 95%CI: 1.49-3.33). In addition, there was an increased risk of larynx cancer per SD increase in haptoglobin (HR: 1.32; 95%CI: 1.21-1.45) and leukocytes (HR: 1.30; 95%CI: 1.20-1.41) (**Table S1**). In contrast, we found a lower risk of cancer in the salivary glands per SD increase in neutrophils in % (HR: 0.41; 95%CI: 0.22-079), PLR (HR: 0.57; 95%CI: 0.34-0.96), and NLR (HR: 0.49; 95%CI: 0.26-0.92), a lower risk of cancer in the nose and middle ear per SD increase in albumin (HR: 0.81; 95%CI: 0.66-0.99), as well as a lower risk of pharynx cancer per SD increase in lymphocytes in % (HR: 0.59; 95%CI: 0.43-0.82).

When analyzing the biomarkers by quartiles, we found that a higher level of haptoglobin was associated with a higher risk of HNC in a concentration-dependent manner (**Table 2**). The multivariable adjusted HR for HNC was 1.16 (95%CI: 1.00-1.34) for haptoglobin level of “1st-2nd quartile”, 1.33 (95%CI: 1.18-1.50) for haptoglobin level of “2nd-3rd quartile”, and 1.79 (95%CI: 1.60-2.00) for haptoglobin level of “above the 3rd quartile”, compared with the reference level of “below the 1st quartile”. The same pattern was noted for squamous cell carcinoma but not adenocarcinoma. Similar results pattern was noted for leukocytes, sedimentation rate, monocytes, monocytes in %, and CAR. A level of lymphocytes in % above the 3^rd^ quartile was associated with a lower risk of squamous cell carcinoma (HR: 0.54; 95%CI: 0.32-0.90).

**Table 2:** Associations between one quartile increase of blood inflammatory biomarkers and the risk of head and neck cancer.

Biomarker	Any head and neck cancer		Squamous cell carcinoma		Adenocarcinoma	
	N of cases/IR	HR (95% CI) ^b^	N of cases/IR	HR (95% CI) ^b^	N of cases/IR	HR (95% CI) ^b^
Haptoglobin (g/l)						
≤1st quartile	590/18.08	ref	471/14.43	ref	31/0.95	ref
1st-2nd quartile	273/22.02	1.16 (1.00-1.34)	224/18.07	**1.20 (1.02-1.41)**	14/1.13	1.03 (0.54-1.98)
2nd-3rd quartile	506/26.25	**1.33 (1.18-1.50)**	424/22.00	**1.40 (1.23-1.60)**	18/0.93	0.87 (0.49-1.56)
>3rd quartile	679/38.80	**1.79 (1.60-2.00)**	597/34.11	**1.97 (1.74-2.23)**	24/1.37	1.19 (0.70-2.04)
CRP (mg/l)						
≤1st quartile	443/24.17	ref	360/19.64	ref	24/1.31	ref
1st-2nd quartile	415/21.69	0.99 (0.86-1.13)	346/18.09	1.02 (0.88-1.19)	16/0.84	0.71 (0.37-1.37)
2nd-3rd quartile	247/25.16	1.09 (0.93-1.28)	216/22.00	1.19 (1.00-1.41)	8/0.81	0.75 (0.33-1.69)
>3rd quartile	405/26.86	1.08 (0.95-1.24)	342/22.68	1.13 (0.97-1.31)	19/1.26	1.01 (0.55-1.85)
Albumin (g/l)						
≤1st quartile	583/26.14	ref	479/21.47	ref	26/1.17	ref
1st-2nd quartile	676/26.54	1.02 (0.91-1.14)	556/21.83	1.01 (0.89-1.15)	28/1.10	0.96 (0.56-1.66)
2nd-3rd quartile	596/24.19	0.99 (0.88-1.11)	512/20.78	1.02 (0.90-1.16)	26/1.06	1.08 (0.62-1.88)
>3rd quartile	459/22.02	1.04 (0.91-1.18)	388/18.61	1.04 (0.90-1.20)	18/0.86	1.11 (0.59-2.08)
Platelets (10^9/l)						
≤1st quartile	206/26.52	ref	170/21.89	ref	7/0.90	
1st-2nd quartile	199/24.11	1.00 (0.82-1.22)	172/20.84	1.05 (0.84-1.30)	3/0.36	0.43 (0.11-1.67)
2nd-3rd quartile	200/23.75	1.03 (0.85-1.26)	164/19.48	1.03 (0.83-1.28)	12/1.43	1.63 (0.63-4.24)
>3rd quartile	206/24.59	1.10 (0.90-1.34)	171/20.41	1.12 (0.90-1.40)	10/1.19	1.56 (0.58-4.16)
Leukocytes (10^9/l)						
≤1st quartile	164/17.66	ref	133/14.32	ref	6/0.65	ref
1st-2nd quartile	221/24.13	**1.36 (1.11-1.66)**	184/20.09	**1.38 (1.11-1.73)**	9/0.98	1.36 (0.47-3.92)
2nd-3rd quartile	188/22.92	**1.33 (1.08-1.64)**	155/18.89	**1.34 (1.06-1.69)**	12/1.46	2.35 (0.88-6.26)
>3rd quartile	260/33.60	**2.00 (1.64-2.43)**	222/28.69	**2.08 (1.67-2.59)**	7/0.90	1.54 (0.51-4.59)
Sedimentation rate (mm/hour)						
≤1st quartile	95/19.73	ref	76/15.78	ref	5/1.04	ref
1st-2nd quartile	105/21.35	1.27 (0.95-1.69)	83/16.88	1.27 (0.92-1.75)	3/0.61	0.60 (0.14-2.56)
2nd-3rd quartile	81/26.06	**1.56 (1.14-2.13)**	69/22.20	**1.71 (1.21-2.41)**	4/1.29	1.26 (0.31-5.03)
>3rd quartile	92/32.41	**1.85 (1.35-2.54)**	81/28.54	**2.12 (1.50-3.00)**	3/1.06	0.94 (0.20-4.51)
Lymphocytes (10^9/l)						
≤1st quartile	34/15.89	ref	31/14.49	ref	2/0.93	ref
1st-2nd quartile	52/22.12	1.42 (0.92-2.19)	39/16.59	1.18 (0.74-1.90)	3/1.28	1.50 (0.25-8.99)
2nd-3rd quartile	54/23.32	1.51 (0.98-2.33)	50/21.59	1.52 (0.97-2.39)	0/0	–
>3rd quartile	52/22.65	1.40 (0.90-2.17)	42/18.30	1.21 (0.75-1.94)	1/0.44	0.53 (0.05-5.88)
Monocytes (10^9/l)						
≤1st quartile	40/15.38	ref	36/13.85	ref	2/0.77	ref
1st-2nd quartile	49/20.68	1.31 (0.86-1.99)	40/16.88	1.18 (0.75-1.86)	1/0.42	0.53 (0.05-5.84)
2nd-3rd quartile	44/20.38	1.29 (0.84-2.00)	33/15.29	1.07 (0.66-1.74)	3/1.39	1.64 (0.26-10.15)
>3rd quartile	59/29.91	**1.82 (1.21-2.75)**	53/26.87	**1.88 (1.22-2.90)**	0/0	–
Neutrophils (10^9/l)						
≤1st quartile	15/13.21	ref	11/9.69	ref	0/0	ref
1st-2nd quartile	26/25.37	1.79 (0.94-3.39)	21/20.49	1.95 (0.93-4.06)	1/0.98	–
2nd-3rd quartile	26/26.30	1.81 (0.95-3.45)	22/22.26	2.07 (0.99-4.32)	0/0	–
>3rd quartile	20/21.29	1.69 (0.86-3.32)	17/18.10	1.99 (0.93-4.26)	2/2.13	–
Lymphocytes (%)						
≤1st quartile	47/21.10	ref	41/18.40	ref	3/1.35	ref
1st-2nd quartile	55/24.42	1.12 (0.75-1.66)	48/21.31	1.08 (0.71-1.65)	1/0.44	0.34 (0.04-3.24)
2nd-3rd quartile	58/23.79	1.12 (0.76-1.65)	49/20.10	1.04 (0.69-1.58)	1/0.41	0.32 (0.03-3.15)
>3rd quartile	34/14.70	0.65 (0.42-1.03)	26/11.24	**0.54 (0.32-0.90)**	1/0.43	0.36 (0.04-3.58)
Monocytes (%)						
≤1st quartile	50/15.96	ref	45/14.36	ref	3/0.96	ref
1st-2nd quartile	60/22.96	1.36 (0.93-1.99)	45/17.22	1.14 (0.75-1.74)	1/0.38	0.38 (0.04-3.67)
2nd-3rd quartile	42/22.18	1.39 (0.92-2.11)	36/19.01	1.34 (0.86-2.09)	2/1.06	0.98 (0.16-6.00)
>3rd quartile	42/26.39	**1.55 (1.02-2.36)**	38/23.87	**1.59 (1.02-2.48)**	0/0	–
Neutrophils (%)						
≤1st quartile	23/20.02	ref	18/15.67	ref	0/0	–
1st-2nd quartile	31/27.44	1.46 (0.84-2.56)	24/21.25	1.49 (0.79-2.82)	1/0.89	–
2nd-3rd quartile	14/15.51	0.88 (0.44-1.72)	12/13.29	1.00 (0.47-2.11)	0/0	–
>3rd quartile	20/21.06	1.22 (0.66-2.25)	18/18.95	1.47 (0.75-2.90)	2/2.11	–
LMR^a^						
≤1st quartile	46/23.83	ref	39/20.21	ref	2/1.04	ref
1st-2nd quartile	50/23.02	1.03 (0.68-1.55)	43/19.80	1.01 (0.65-1.57)	1/0.46	0.46 (0.04-5.20)
2nd-3rd quartile	58/24.33	1.02 (0.68-1.53)	47/19.71	0.93 (0.59-1.44)	1/0.42	0.47 (0.04-5.41)
>3rd quartile	34/13.78	0.62 (0.39-0.98)	31/12.57	0.64 (0.39-1.04)	1/0.41	0.49 (0.04-5.77)
PLR^a^						
≤1st quartile	55/26.86	ref	43/21.00	ref	1/0.49	ref
1st-2nd quartile	47/21.73	0.85 (0.57-1.26)	43/19.88	1.01 (0.65-1.55)	1/0.46	0.96 (0.06-15.31)
2nd-3rd quartile	46/20.94	0.87 (0.58-1.29)	38/17.30	0.94 (0.60-1.46)	1/0.46	0.94 (0.06-15.28)
>3rd quartile	35/17.27	0.70 (0.45-1.08)	32/15.79	0.84 (0.52-1.34)	2/0.99	2.02 (0.18-22.94)
NLR^a^						
≤1st quartile	19/16.82	ref	14/12.40	ref	0/0.00	–
1st-2nd quartile	24/21.83	1.43 (0.77-2.66)	20/18.19	1.70 (0.83-3.48)	0/0.00	–
2nd-3rd quartile	20/18.76	1.20 (0.63-2.32)	15/14.07	1.28 (0.59-2.76)	1/0.94	–
>3rd quartile	24/24.42	1.65 (0.88-3.08)	22/22.38	2.20 (1.08-4.46)	2/2.03	–
CAR^a^						
≤1st quartile	399/22.91	ref	322/18.49	ref	23/1.32	ref
1st-2nd quartile	329/22.27	1.09 (0.94-1.26)	272/18.41	1.13 (0.96-1.33)	12/0.81	0.71 (0.35-1.44)
2nd-3rd quartile	357/23.08	1.09 (0.95-1.26)	310/20.05	**1.20 (1.02-1.40)**	11/0.71	0.61 (0.29-1.26)
>3rd quartile	414/27.13	**1.16 (1.01-1.33)**	346/22.68	**1.21 (1.04-1.41)**	20/1.31	1.01 (0.55-1.85)

a Logarithmic transformation (log2) was used for LMR, PLR, NLR, and CAR.

b Analyses were adjusted for age, sex, fasting status, occupational status, and country of birth.

CRP, C-reactive protein; LMR, lymphocyte-to-monocyte ratio; PLR, platelet-to-lymphocyte ratio; NLR, neutrophil-to-lymphocyte ratio; CAR, C-reactive protein-to-albumin ratio.

Bold text indicates statistical significance at P<0.05.

Using current clinical references, we classified the participants as having normal or abnormal levels of the biomarkers and found that an increased risk of HNC was associated with a high level of haptoglobin (≥1.4 mg/l), leukocytes (≥10 x 10^9/l), and sedimentation rate (≥10 mm/h) (**Table 3**). We also found that a decreased risk of HNC was associated with a low level of monocytes (≤0.2 x 10^9/l) and a high level of lymphocytes in % (≥42.00%). These associations were observed for squamous cell carcinoma but not adenocarcinoma. A high level of albumin (≥40 g/l) was associated with a lower risk of squamous cell carcinoma. No clear association was observed for adenocarcinoma except for a positive association with a high level of neutrophils in % (≥70.00%).

**Table 3:** Associations between clinically abnormal levels of blood inflammatory biomarkers and the risk of head and neck cancer.

Biomarker		Any head and neck cancer		Squamous cell carcinoma		Adenocarcinoma	
		N of cases/IR	HR (95% CI) ^a^	N of cases/IR	HR (95% CI) ^a^	N of cases/IR	HR (95% CI) ^a^
Haptoglobin (g/l)							
<1.4		1555/22.10	ref	1269/18.04	ref	68/0.97	ref
≥1.4		493/43.05	**1.65 (1.49-1.83)**	447/39.04	**1.83 (1.64-2.04)**	19/1.66	1.51 (0.90-2.52)
CRP (mg/l)							
<10		1262/23.84	ref	1061/20.04	ref	54/1.02	ref
≥10		248/26.34	1.03 (0.90-1.18)	203/21.56	1.00 (0.86-1.17)	13/1.38	1.27 (0.69-2.34)
Albumin (g/l)							
<40		213/29.70	ref	180/25.10	ref	8/1.12	ref
≥40		2101/24.41	0.87 (0.75-1.00)	1755/20.39	**0.84 (0.71-0.98)**	90/1.05	1.09 (0.52-2.26)
Platelets (10^9/l)							
≤100		1/22.33	0.88 (0.12-6.26)	1/22.33	1.05 (0.15-7.50)	0/0	–
100~300		604/24.76	ref	505/20.70	ref	22/0.90	ref
≥300		206/24.59	1.09 (0.92-1.28)	171/20.41	1.10 (0.92-1.31)	10/1.19	1.52 (0.71-3.27)
Leukocytes (10^9/l)							
<10		764/23.51	ref	633/19.48	ref	33/1.02	ref
≥10		69/36.40	**1.70 (1.32-2.18)**	61/32.18	**1.79 (1.37-2.34)**	1/0.53	0.59 (0.08-4.35)
Sedimentation rate (mm/h)							
<15		312/22.41	ref	257/18.46	ref	12/0.86	ref
≥15		61/34.64	**1.51 (1.13-2.01)**	52/29.53	**1.59 (1.16-2.18)**	3/1.70	1.84 (0.49-6.95)
Lymphocytes (10^9/l)							
≤0.80		3/17.45	0.80 (0.26-2.51)	2/11.63	0.63 (0.16-2.55)	0/0	–
0.80~3.5		180/21.44	ref	154/18.34	ref	6/0.43	ref
≥3.5		9/16.87	0.71 (0.35-1.44)	6/11.24	0.52 (0.21-1.26)	0/0	–
Monocytes (10^9/l)							
≤0.20		23/12.56	**0.56 (0.36-0.87)**	2/10.92	**0.58 (0.36-0.92)**	2/1.09	2.04 (0.37-11.30)
0.20~0.80		158/23.11	ref	132/19.30	ref	4/0.58	ref
≥0.80		11/25.53	1.01 (0.53-1.92)	10/23.21	1.10 (0.56-2.18)	0/0	–
Neutrophils (10^9/l)							
≤1.80		0/0	–	0/0	–	0/0	–
1.80~7.00		82/22.13	ref	66/17.81	ref	3/0.45	ref
≥7.00		5/23.30	1.26 (0.51-3.12)	5/23.30	1.61 (0.64-4.02)	0/0	–
Lymphocytes (%)							
≤18.00		9/23.69	1.12 (0.57-2.19)	8/21.06	1.19 (0.58-2.43)	1/2.63	4.32 (0.47-39.53)
18.00~42.00		157/23.05	ref	134/19.67	ref	4/0.59	ref
≥42.00		28/13.72	**0.56 (0.37-0.86)**	22/10.78	**0.50 (0.31-0.80)**	1/0.49	0.92 (0.10-8.35)
Monocytes (%)							
≤2.00		18/14.75	0.70 (0.43-1.14)	15/12.29	0.70 (0.41-1.19)	2/2.63	3.19 (0.58-17.56)
2.00~10.00		154/21.85	ref	128/18.16	ref	4/0.57	ref
≥10.00		22/22.83	1.02 (0.65-1.60)	21/21.79	1.19 (0.74-1.90)	0/0	–
Neutrophils (%)							
≤40.00		1/5.03	0.23 (0.03-1.67)	0/0	–	0/0	–
40.00~70.00		80/22.89	ref	67/19.17	ref	1/0.29	ref
≥70.00		7/16.00	0.80 (0.37-1.73)	5/11.43	0.70 (0.28-1.75)	2/4.57	**19.26 (1.63-227.37)**

a Analyses were adjusted for age, sex, fasting status, occupational status, and country of birth.

CRP, C-reactive protein; LMR, lymphocyte-to-monocyte ratio; PLR, platelet-to-lymphocyte ratio; NLR, neutrophil-to-lymphocyte ratio; CAR, C-reactive protein-to-albumin ratio.

Bold text indicates statistical significance at P<0.05.

### 3.2 Nested case-control study analysis

**Figure 2:**
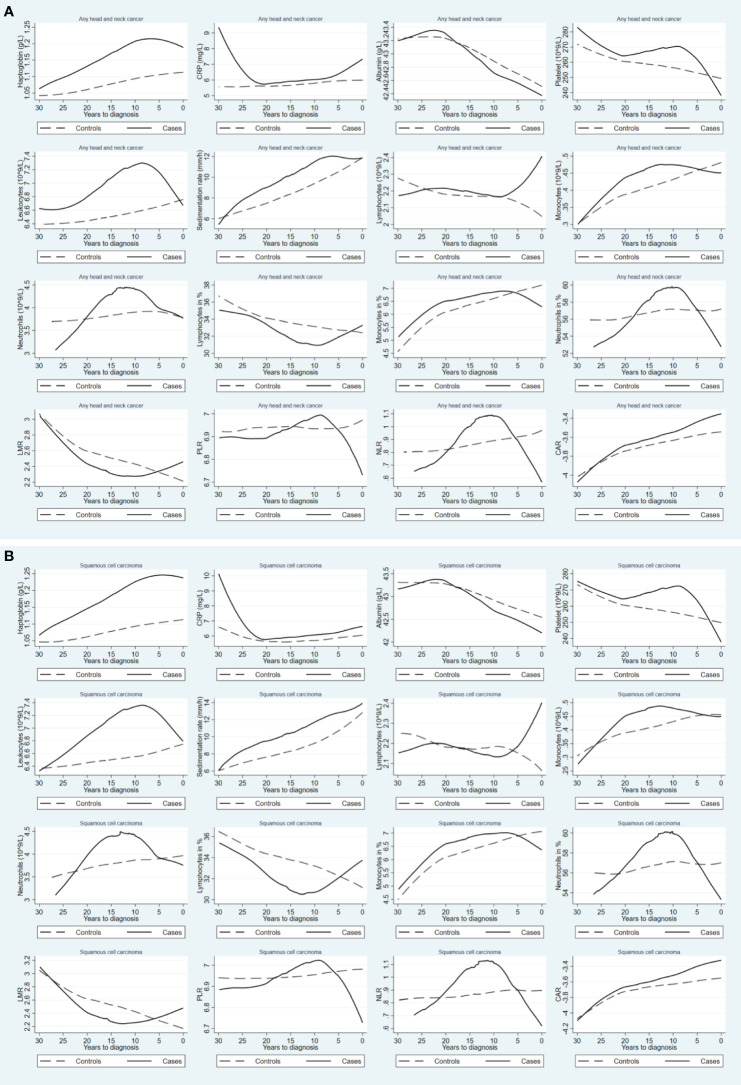
Mean concentrations of blood inflammatory biomarkers during the 30-year period prior to diagnosis of any head and neck cancer **(A)** and squamous cell carcinoma **(B)**, comparing patients with head and neck cancer (solid line) to their matched controls (dashed line). CRP: C-reactive protein; LMR: lymphocyte-to-monocyte ratio; PLR: platelet-to-lymphocyte ratio; NLR: neutrophil-to-lymphocyte ratio; CAR: C reactive protein-to-albumin ratio.

**Figure 3:**
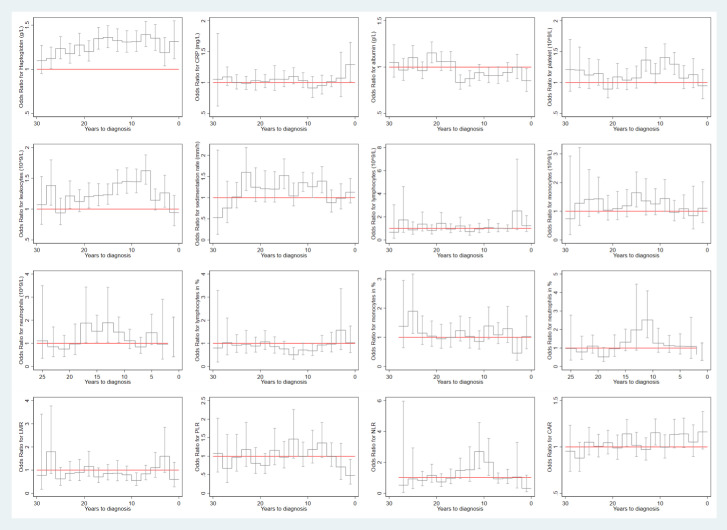
Risk of any head and neck cancer in relation to the level of biomarkers during each 2-year time window of the 30-year period prior to cancer diagnosis. CRP: C-reactive protein; LMR: lymphocyte-to-monocyte ratio; PLR: platelet-to-lymphocyte ratio; NLR: neutrophil-to-lymphocyte ratio; CAR: C reactive protein-to-albumin ratio.

The mean concentrations of the studied biomarkers during the 30-year period prior to the index date for both cases and matched controls are shown in **Figure 2**. During the 30-year period before diagnosis, individuals with HNC exhibited constantly elevated levels of haptoglobin, leukocytes, sedimentation rate, monocytes, and monocytes in %, but decreased levels of lymphocytes in % and LMR, compared to controls. Similar findings were observed for squamous cell carcinoma (**Figure 2B**). **Figure 3** shows a positive association between a higher level of haptoglobin, leukocytes, sedimentation rate, and monocytes and a higher risk of HNC, as well as a negative association between lymphocytes in % and LMR and the risk of HNC, in most of the two-year time windows prior to cancer diagnosis.

## 4 Discussion

Based on a cohort study of more than half a million participants with up to 35 years of follow-up, our study showed that individuals with a higher blood level of haptoglobin, leukocytes, sedimentation rate, and monocytes had an elevated risk of HNC, whereas those with a higher blood level of lymphocytes in % and LMR had a lower risk of HNC. The associations were mainly noted for squamous cell carcinoma, as well as for cancers in the lip and oral cavity, larynx, and pharynx. We also found that individuals with HNC exhibited higher-than-expected levels of haptoglobin, leukocytes, sedimentation rate, and monocytes, as well as lower-than-expected levels of lymphocytes in % and LMR, during the 30-year period prior to cancer diagnosis.

In our literature review, we identified five case-control studies (20–24) that assessed the role of haptoglobin in HNC risk. Four of these studies found a higher level of haptoglobin in patients with oral squamous cell carcinoma (OSCC) (21–23) or laryngeal cancer (24), compared to controls, in agreement with our finding. One study found, however, a lower level of haptoglobin among patients with OSCC than controls (20). The inconsistent results may be due to different reasons, including potentially different measurements of haptoglobin, as patients with HNC might have a higher level of haptoglobin α2 chain but a lower level of haptoglobin α1 chain (62). In terms of leukocytes, we identified one cohort study (12) and seven case-control studies (12, 26, 31–34, 36, 38). The cohort study reported a positive association between the level of leukocytes and the risk of cancer in the oral cavity and pharynx (12), in line with our finding. Out of the seven case-control studies, four studies found elevated levels of leukocytes in patients with nasopharyngeal carcinoma (31), laryngeal cancer (33), OSCC (26), and head and neck squamous cell carcinoma (HNSCC) (38), while the rest reported no difference between patients with HNC and controls (32, 34, 36). In terms of sedimentation rate, we identified one cohort study (37) and one case-control study (18). The cohort study reported that an elevated sedimentation rate was associated with an increased risk of cancers in the lip and oral cavity, the salivary glands, and the pharyngeal cavity and tonsils, a decreased risk of larynx cancer, and no altered risk of cancer in the tongue (37). The case-control study reported an elevated levels of sedimentation rate in patients with HNSCC compared to controls (18). Finally, we identified four case-control studies on monocytes and risk of HNC (26, 29, 31, 38). Three of the four studies reported an elevated level of monocytes in patients with nasopharyngeal cancer (31), OSCC (26), and HNSCC (38), compared to controls, in agreement with our finding. One study reported, however, no difference between patients and controls (29). Taken together, the existing studies, mostly cross sectional in nature, and the present study, using a time-to-event analysis of prospectively collected data, jointly suggest a positive association between blood levels of haptoglobin, leukocytes, sedimentation rate, and monocytes and risk of HNC, especially squamous cell carcinoma.

When it comes to lymphocytes, we identified five case-control studies (26, 29, 31–33) in our literature review. Three studies reported a lower level of lymphocytes in patients with cancer in the salivary glands (29), nasopharyngeal cancer (31), or cancer in the oral cavity (32), while two reported an elevated level of lymphocytes in patients with laryngeal cancer (33) or OSCC (26), compared to controls. Although we did not find statistically significant results for total count of lymphocytes, we found a higher level of lymphocytes in % or LMR to be associated with a lower risk of HNC. More studies are therefore needed to further understand the role of lymphocytes in the risk of NPC, both in absolute and relative quantities.

Apart from the above six biomarkers, we did not find clear results for any of the other six biomarkers studied. For instance, we identified two cohort studies (9, 12) and nine case-control studies (10, 11, 13–19) that assessed the association of CRP with the risk of HNC. The cohort studies found an increased risk of HNC in relation to a higher level of CRP (9, 12). Eight case-control studies found a higher level of CRP in patients with HNC, compared to controls, whereas one study found no difference between cases and controls (16). The null finding of CRP in the present study might be since we did not measure high sensitivity CRP as it was not available in Sweden in the recruitment period of the AMORIS Cohort. Our study did not reveal clear association between albumin level and risk of HNC. To our best knowledge, three case-control studies have examined albumin in HNC so far (25–27). Two studies reported a lower level of albumin in patients with OSCC (25, 26) whereas the other reported a lower level of albumin in patients with oral cancer (27). We found a positive association between the level of platelets and the risk of squamous cell carcinoma but not any HNC or adenocarcinoma in the present study. In the literature review, we found ten case-control studies in this regard (26, 28–36). Three reported an elevated level of platelets in patients with OSCC (26, 28) or nasopharyngeal carcinoma (31), when compared to controls, whereas the other seven did not find a difference between cases and controls (29, 30, 32–36). Finally, we did not find neutrophils to be associated with the risk of HNC in the present study. In the literature review, we identified seven case-control studies (26, 29, 31–33, 36, 38), including five studies that reported an elevated count of neutrophils in patients with cancer in the salivary glands (29), nasopharyngeal cancer (31), laryngeal cancer (33), OSCC (26), or HNSCC (38), compared to controls, as well as two studies reporting no difference between patients with cancer in the oral cavity (32) or laryngeal squamous cell carcinoma (LSCC) (36) and controls. In terms of PLR, ten case-control studies were identified in the literature review (28–30, 39–45). Eight studies reported an elevated level of PLR in patients with LSCC (39), nasopharyngeal cancer (40), cancer in the salivary glands (29, 44), larynx and hypopharynx cancer (42), parotid cancer (30), HNC (43), and laryngeal cancer (45), whereas two reported no statistically significant difference between patients with OSCC (28) or parotid cancer (41) and controls. In terms of NLR, 18 case-control studies were identified in our literature review (29, 30, 32–34, 36, 38–41, 43–50), including 17 studies reporting a higher level of NLR in patients with HNC, compared to controls, and one study reporting no difference between patients with laryngeal cancer and controls (47). Finally, like lymphocytes in % and LMR discussed above, there is currently no other study on monocytes in %, neutrophils in %, or CAR concerning the risk of HNC. Regardless, for all biomarkers discussed herein, the contrasting findings between the present study and the previous studies might have importantly been attributed to reverse causation, as the previous studies mostly measured biomarker levels following a diagnosis of HNC. For instance, one might speculate that inflammation level (e.g., CRP, neutrophils, and NLR) might increase whereas albumin level might decrease following a cancer diagnosis, because of cancer biology, cancer treatment, or both.

A link between blood inflammatory biomarkers and the risk of HNC is biologically plausible, as chronic inflammation has been hypothesized to promote HNC development and progression (6, 63). For instance, recent studies have shown that haptoglobin is a highly complex glycoprotein containing four N-glycosylation sites and is characterized by highly sialylated N-glycans (64). This complex glycosylation pattern makes haptoglobin particularly susceptible to altered glycosylation, which might influence tumor development and progression (65). Our findings suggest that blood inflammatory markers are primarily associated with HNSCC instead of adenocarcinoma. One reason might be the different molecular pathogenesis between squamous cell carcinoma and adenocarcinoma. Genetic instability is more commonly observed in patients with HNSCC, compared to adenocarcinoma. For example, TP53 mutations are present in 70.4% of squamous cell carcinoma but only in 28% of adenocarcinoma (66, 67) and inflammation has been suggested to increase DNA damage and mutations via generating reactive oxygen species (68). Another reason is the difference in risk factors between squamous cell carcinoma and adenocarcinoma. For instance, HPV infection has been suggested as a risk factor for HNSCC, especially oral and oropharyngeal squamous cell carcinoma (2), but is very rare in head and neck adenocarcinoma (69). Further, inflammation-related cytokines are known to regulate HPV proliferation and modulate its oncogenes E7 and E8 in cervical epithelial cells (70). Similarly, inflammation was shown to be associated with HPV infection status of tongue squamous cell carcinoma (71). Finally, the limited number of individuals with adenocarcinoma might also have contributed to a lack of statistical power in the analyses of adenocarcinoma.

This study has several strengths. First, the study has a large sample size, a population-based design, prospectively collected data on inflammatory biomarkers, and a long and complete follow-up, reducing the risk of selection bias due to selective study participation or loss to follow-up as well as random error. Second, we estimated the associations by histopathology type and site of HNC to investigate whether the roles of the studied biomarkers might differ by these factors. Third, to minimize the possibility of reverse causation, we excluded the initial five years of follow-up from the analysis. Additionally, we conducted a nested case-control study to estimate the temporal trends of the studied biomarkers during 30-year period prior to cancer diagnosis, which corroborated findings of the time-to-event analysis. For example, specific biomarkers, such as haptoglobin and leukocytes, which were shown to have positive associations with the risk of HNC in the time-to-event analysis, demonstrated higher-than-expected levels during the 30-year period prior to cancer diagnosis among individuals with HNC. Finally, a sensitivity analysis was performed to address potential indication bias by restricting the analysis of biomarker measurements obtained during an occupational health check-up. There are also limitations in this study. Given the register-based nature of the present study, the main limitation is the inability to study the contributors to the altered blood inflammatory biomarkers. For instance, tobacco smoking has been associated with an altered level of inflammatory biomarkers (72), including haptoglobin, CRP, and count of leukocytes (73). As a result, our findings on these biomarkers might indicate an indirect effect of tobacco smoking on the risk of HNC. Further, the nested case-control analysis was based on a well-defined cohort with complete follow-up and included cases and controls individually matched by age, sex, and calendar period of recruitment to the AMORIS Cohort, using the method of incidence sampling. However, although the cases and controls had identical time window for assessment of biomarker levels given the design, the cases and controls did not always have the same number of repeated measurements for each biomarker during the time window. It is possible that individuals with an upcoming cancer diagnosis had more measurements, compared to others. This will however most likely affect the result patten noted during the last few years, but not the entire 30 years, before cancer diagnosis.

## 5 Conclusion

In this large cohort study with prospectively measured blood inflammatory biomarkers, we found a positive association of haptoglobin, leukocytes, sedimentation rate, and monocytes, and an inverse association of lymphocytes in percentage and LMR, with the risk of HNC. Our findings therefore provide solid evidence supporting the presence of alterations in blood inflammatory biomarkers during the decades before the diagnosis of HNC.

## Data availability statement

The datasets presented in this article are not readily available because Data and materials used in the present study are not publicly available due to EU and Swedish regulations. Please contact the Steering Group for AMORIS Cohort for more information and potential collaborations (https://ki.se/imm/amoris). Requests to access the datasets should be directed to https://ki.se/imm/amoris.

## Ethics statement

The studies involving humans were approved by Swedish Ethical Review Authority. The studies were conducted in accordance with the local legislation and institutional requirements. Written informed consent for participation was not required from the participants or the participants’ legal guardians/next of kin in accordance with the national legislation and institutional requirements.

## Author contributions

YY: Conceptualization, Data curation, Formal Analysis, Investigation, Writing – original draft. YL: Data curation, Investigation, Writing – original draft, Conceptualization. FS: Investigation, Methodology, Visualization, Writing – review & editing. MF: Investigation, Methodology, Resources, Writing – review & editing, Project administration. NH: Investigation, Methodology, Resources, Writing – review & editing, Project administration. FF: Funding acquisition, Writing – review & editing, Resources, Investigation, Methodology. ZZ: Investigation, Supervision, Writing – review & editing, Funding acquisition, Project administration, Conceptualization, Methodology. QL: Supervision, Writing – review & editing, Investigation, Conceptualization, Methodology, Visualization, Formal Analysis.
