# Supplementary material for: Risk of head and neck cancer in relation to blood inflammatory biomarkers in the Swedish AMORIS cohort
Source: Front Immunol. 2023 Oct 9;14:1265406. doi: 10.3389/fimmu.2023.1265406 (PMC10590876; doi:10.3389/fimmu.2023.1265406)
Supplement: Supplementary file 1 [file Image_1.pdf]

Biomarker	Head and neck cancer N of cases/IR	Head and neck cancer HR (95% CI)[b]	Squamous cell carcinoma N of cases/IR	Squamous cell carcinoma HR (95% CI)[b]	Adenocarcinoma N of cases/IR	Adenocarcinoma HR (95% CI)[b]
Haptoglobin	1747/25.26	1.27 (1.22−1.32)	1464/21.17	1.30 (1.24−1.36)	77/1.11	1.24 (1.02−1.52)
CRP	1233/24.72	1.00 (0.94−1.05)	1033/20.71	1.00 (0.94−1.06)	56/1.12	0.91 (0.52−1.58)
Albumin	1921/25.15	0.98 (0.94−1.04)	1603/20.99	1.00 (0.95−1.06)	85/1.11	1.00 (0.78−1.27)
Platelet	505/26.22	1.13 (1.04−1.24)	416/21.60	1.13 (1.03−1.25)	25/1.30	1.35 (1.00−1.81)
Leukocytes	523/25.56	1.31 (1.20−1.42)	430/21.01	1.34 (1.23−1.46)	26/1.27	1.30 (0.90−1.89)
Sedimentation rate	194/22.35	1.26 (1.11−1.44)	159/18.32	1.33 (1.17−1.51)	8/0.92	0.65 (0.13−3.14)
Lymphocytes	110/21.78	1.04 (0.67−1.63)	93/18.41	0.95 (0.56−1.60)	4/0.79	1.13 (0.15−8.52)
Monocytes	110/21.78	1.33 (0.99−1.78)	93/18.32	1.44 (1.06−1.96)	4/0.79	0.19 (0.02−2.11)
Neutrophils	50/23.25	1.20 (0.92−1.57)	42/19.53	1.29 (0.98−1.69)	1/0.47	1.54 (0.88−2.71)
Lymphocytes (%)	112/21.70	0.81 (0.66−0.99)	95/18.40	0.73 (0.58−0.91)	4/0.77	1.05 (0.38−2.94)
Monocytes (%)	112/21.70	1.13 (0.94−1.36)	95/18.41	1.18 (0.97−1.44)	4/0.77	0.36 (0.10−1.30)
Neutrophils (%)	51/23.32	1.00 (0.75−1.33)	43/19.66	1.11 (0.81−1.53)	1/0.46	1.00 (1.00−1.00)
LMR[a]	107/21.61	0.80 (0.66−0.98)	92/18.58	0.79 (0.63−0.98)	3/0.61	1.29 (0.47−3.56)
PLR[a]	103/22.77	0.99 (0.80−1.22)	88/19.45	1.03 (0.82−1.30)	4/0.88	1.09 (0.38−3.10)
NLR[a]	50/22.02	1.13 (0.83−1.52)	42/18.50	1.28 (0.92−1.77)	1/0.44	8.18 (0.37−180)
CAR[a]	1223/24.22	1.04 (0.98−1.09)	1021/20.22	1.05 (0.99−1.12)	55/1.09	0.98 (0.75−1.27)

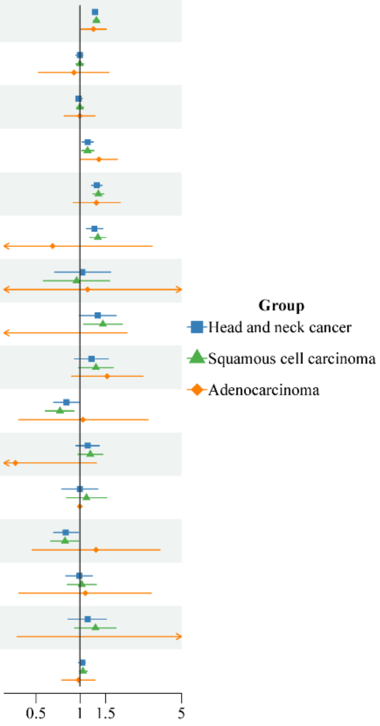

Supplementary Figure 1. The association between one standard deviation increase of blood inflammatory biomarkers and the risk of head and neck cancer, analysis restricted to biomarker measurement obtained during an occupational health check-up. CRP: C-reactive protein; LMR: lymphocyte-to-monocyte ratio; PLR: platelet-to-lymphocyte ratio; NLR: neutrophil-to-lymphocyte ratio; CAR: C-reactive protein-to-albumin ratio; IR: Incidence rates; HR: hazard ratios; CI: confidence intervals.[a] Logarithmic transformation (log2) was used for LMR, PLR, NLR, and CAR. [b] Analyses were adjusted for age, sex, fasting status, occupational status, and country of birth.
