# Supplementary material for: Risk of head and neck cancer in relation to blood inflammatory biomarkers in the Swedish AMORIS cohort
Source: Front Immunol. 2023 Oct 9;14:1265406. doi: 10.3389/fimmu.2023.1265406 (PMC10590876; doi:10.3389/fimmu.2023.1265406)
Supplement: Supplementary file 2 [file Table_1.docx]

**Table S1.** Associations one standard deviation increase of blood inflammatory biomarkers and the risk of head and neck cancer, analysis by cancer site

| **Biomarker** | **Cancer in the lip and oral cavity** | | **Cancer in the salivary glands** | | **Pharynx cancer** | | **Cancer in nose and middle ear** | | **Larynx cancer** | |
| --- | --- | --- | --- | --- | --- | --- | --- | --- | --- | --- |
|  | **N of cases** | **HR (95% CI)** ^b^ | **N of cases** | **HR (95% CI)** ^b^ | **N of cases** | **HR (95% CI)** ^b^ | **N of cases** | **HR (95% CI)** ^b^ | **N of cases** | **HR (95% CI)** ^b^ |
| Haptoglobin | 896 | **1.21 (1.14-1.29)** | 139 | 1.04 (0.87-1.24) | 525 | **1.33 (1.24-1.42)** | 109 | 1.14 (0.95-1.37) | 318 | **1.32 (1.21-1.45)** |
| CRP | 668 | 1.0 (0.93-1.08) | 109 | 0.86 (0.54-1.39) | 403 | 0.93 (0.78-1.12) | 75 | 0.91 (0.58-1.45) | 217 | 1.04 (0.96-1.12) |
| Albumin | 1008 | 1.07 (0.99-1.15) | 160 | 1.12 (0.94-1.34) | 615 | 0.91 (0.84-1.00) | 117 | **0.81 (0.66-0.99)** | 348 | 0.92 (0.82-1.04) |
| Platelet | 362 | **1.12 (1.01-1.24)** | 53 | 0.80 (0.58-1.10) | 206 | 1.06 (0.92-1.23) | 36 | 1.08 (0.77-1.52) | 125 | 0.99 (0.82-1.20) |
| Leukocytes | 372 | **1.17 (1.08-1.28)** | 54 | 1.10 (0.82-1.48) | 214 | **1.28 (1.19-1.36)** | 36 | 1.03 (0.69-1.55) | 127 | **1.30 (1.20-1.41)** |
| Sedimentation rate | 165 | **1.18 (1.02-1.35)** | 20 | 1.10 (0.71-1.71) | 89 | **1.23 (1.04-1.46)** | 23 | 0.42 (0.14-1.31) | 63 | 1.22 (1.00-1.50) |
| Lymphocytes | 91 | 1.00 (0.60-1.65) | 10 | 1.55 (0.90-2.66) | 47 | 0.74 (0.32-1.67) | 9 | 0.40 (0.05-3.25) | 28 | 1.33 (0.76-2.30) |
| Monocytes | 91 | 1.26 (0.89-1.78) | 10 | 1.08 (0.39-3.04) | 47 | 1.50 (0.97-2.33) | 9 | 0.87 (0.26-2.98) | 28 | 1.59 (0.95-2.69) |
| Neutrophils | 37 | 1.16 (0.85-1.58) | 7 | 0.42 (0.13-1.38) | 22 | **1.57 (1.20-2.04)** | 5 | 0.51 (0.13-2.06) | 13 | 0.91 (0.47-1.75) |
| Lymphocytes (%) | 92 | 0.88 (0.70-1.10) | 10 | 1.68 (0.98-2.86) | 48 | **0.59 (0.43-0.82)** | 9 | 1.05 (0.53-2.09) | 28 | 0.98 (0.66-1.46) |
| Monocytes (%) | 92 | 1.10 (0.89-1.37) | 10 | 0.99 (0.52-1.87) | 48 | 1.12 (0.83-1.51) | 9 | 1.27 (0.66-2.44) | 28 | 1.40 (0.98-2.00) |
| Neutrophils (%) | 38 | 1.08 (0.76-1.51) | 7 | **0.41 (0.22-0.79)** | 22 | **2.10 (1.30-3.41)** | 5 | 0.71 (0.29-1.71) | 13 | 0.77 (0.45-1.32) |
| LMR^a^ | 89 | 0.84 (0.67-1.06) | 9 | 0.85 (0.42-1.74) | 47 | 0.77 (0.56-1.07) | 8 | 0.66 (0.30-1.48) | 28 | 0.80 (0.53-1.20) |
| PLR^a^ | 84 | 0.88 (0.70-1.10) | 9 | **0.57 (0.34-0.96)** | 46 | 1.21 (0.87-1.67) | 9 | 1.14 (0.55-2.35) | 28 | 0.79 (0.54-1.16) |
| NLR^a^ | 37 | 1.11 (0.77-1.58) | 7 | **0.49 (0.26-0.92)** | 22 | **2.23 (1.49-3.33)** | 5 | 0.85 (0.33-2.19) | 13 | 0.77 (0.44-1.34) |
| CAR^a^ | 655 | 1.07 (0.99-1.15) | 113 | 0.96 (0.79-1.16) | 398 | 1.07 (0.97-1.18) | 75 | 0.86 (0.68-1.08) | 220 | 1.04 (0.91-1.18) |
| ^a^ Logarithmic transformation (log2) was used for LMR, PLR, NLR, and CAR.  ^b^ Analyses were adjusted for age, sex, fasting status, occupational status, and country of birth.  CRP: C-reactive protein; LMR: lymphocyte-to-monocyte ratio; PLR: platelet-to-lymphocyte ratio; NLR: neutrophil-to-lymphocyte ratio; CAR: C-reactive protein/albumin ratio | | | | | | | | | | |
